# Hamstring, bone-patellar tendon-bone, quadriceps and peroneus longus tendon autografts for primary isolated posterior cruciate ligament reconstruction: a systematic review

**DOI:** 10.1093/bmb/ldac010

**Published:** 2022-04-22

**Authors:** Filippo Migliorini, Andrea Pintore, Gianluca Vecchio, Francesco Oliva, Frank Hildebrand, Nicola Maffulli

**Affiliations:** Department of Orthopaedic, Trauma, and Reconstructive Surgery, RWTH Aachen University Hospital, Aachen 52064, Germany; Department of Orthopaedics, Surgery and Dentistry, University of Salerno, Via S. Allende, Baronissi, Salerno (SA) 84081, Italy; Department of Orthopaedics, Surgery and Dentistry, University of Salerno, Via S. Allende, Baronissi, Salerno (SA) 84081, Italy; Department of Orthopaedics, Surgery and Dentistry, University of Salerno, Via S. Allende, Baronissi, Salerno (SA) 84081, Italy; Department of Orthopaedic, Trauma, and Reconstructive Surgery, RWTH Aachen University Hospital, Aachen 52064, Germany; Department of Orthopaedics, Surgery and Dentistry, University of Salerno, Via S. Allende, Baronissi, Salerno (SA) 84081, Italy; Queen Mary University of London, Barts and the London School of Medicine and Dentistry, Centre for Sports and Exercise Medicine, Mile End Hospital, 275 Bancroft Road, London E1 4DG, UK; School of Pharmacy and Bioengineering, Keele University Faculty of Medicine, Thornburrow Drive, 01782 Stoke on Trent, UK

**Keywords:** posterior cruciate ligament, autograft, quadriceps, bone-patellar tendon-bone, hamstring, peroneus longus

## Abstract

**Introduction:**

Several autografts are available to reconstruct the posterior cruciate ligament (PCL).

**Source of data:**

Current scientific literature published in PubMed, Google scholar, Embase and Scopus.

**Areas of agreement:**

Hamstring, bone-patellar tendon-bone (BPTB), quadriceps and peroneus longus (PLT) are the most common tendon autografts used for primary isolated PCL reconstruction.

**Areas of controversy:**

The optimal tendon source for PCL reconstruction remains nevertheless debated. Identifying the most suitable tendon autograft could assist the surgeon during primary PCL reconstruction.

**Growing points:**

The present study compared the outcome of PCL reconstruction using hamstring, BPTB, quadriceps and PLT autografts. The focus was on patient-reported outcome measures (PROMs), joint laxity, range of motion and complications.

**Areas timely for developing research:**

All autografts are viable options for PCL reconstruction, with BTB and hamstring autografts demonstrating superior PROMs. However, further clinical investigations are required to determine the ideal autograft construct.

## Introduction

The posterior cruciate ligament (PCL) is the primary restraint to posterior tibial translation.^1^ The incidence of PCL rupture ranges from 1 to 40% of all acute knee injuries.^2^ PCL tears typically occur during high-energy trauma, such as motor vehicle accidents or fall on the knee with the foot in a plantar flexed position.^3^ PCL tears are diagnosed by physical examination and magnetic resonance imaging. Symptomatic PCL ruptures with posterior displacement >8 mm and instability may be managed by surgical reconstruction.^4–7^ Several tendon autografts for PCL reconstruction have been employed, such as the hamstring, bone-patellar tendon-bone (BPTB), quadriceps and peroneus longus tendon (PLT).^8–14^ Hamstring autografts are the most commonly used tendons for PCL reconstruction.^12^^,^^15^^,^^16^ BPTB has been also employed for PCL reconstruction, with fast incorporation, quick return to preinjury activity levels and low risk of graft rupture.^17–19^ Quadriceps tendon autograft represents another valuable option for PCL reconstruction, demonstrating high level of activity after surgery.^20–22^ PLT autografts have been employed for PCL reconstruction with satisfying clinical outcomes.^23^ The optimal tendon source for PCL reconstruction remains nevertheless debated. Identifying the most suitable tendon autograft could assist the surgeon during primary PCL reconstruction. The present study compared the outcome of PCL reconstruction using hamstring, BPTB, quadriceps and PLT autografts. The focus was on patient-reported outcome measures (PROMs), joint laxity, range of motion (ROM) and complications.

## Material and methods

### Search strategy

This systematic review was conducted in accordance with the Preferred Reporting Items for Systematic Reviews and Meta-Analyses (PRISMA).^24^ The PICOT algorithm guided the initial search:

- P (population): PCL tears;
- I (intervention): primary isolated PCL reconstruction;
- C (comparison): hamstring, BPTB, quadriceps, PLT autografts;
- O (outcomes): PROMs, ROM, laxity, complications;
- T (Timing): > 12 months of follow-up.

### Literature search

Two authors (F.M. & A.P.) independently performed the literature search in April 2021. The following databases were accessed: PubMed, Google Scholar, Embase and Scopus. The following keywords were used for the search: ‘posterior cruciate ligament, autograft, graft, tendon, quadriceps, bone-patellar tendon-bone, hamstring, reconstruction, peroneus longus, BPTB, PCL, ligament, Lysholm, PROM, patient reported outcome measures, laxity, stability, instability, range of motion, anterior knee pain, reoperation, revision, pain’. Titles and abstracts were screened by the same authors in a separate fashion. If the abstract matched the topic of interest, the full text of the article was accessed. The bibliographies were screened to identify additional articles. Disagreements were resolved by a third author (^**^).

### Eligibility criteria

All the clinical studies investigating the outcome of PCL reconstruction using an autograft were accessed. Only studies that clearly stated the source of the graft were included. The autografts of interest were hamstring, BPTB, quadriceps and PLT. Studies reporting data on other autografts, allografts or synthetic grafts were excluded. Given the authors’ language abilities, articles in English, German, Italian and French were eligible. Comments, reviews, letters, notes, protocols, editorials, guidelines and registries were not considered. Computational, animal, biomechanical and cadaveric studies were also not eligible. Only studies reporting data from a minimum of 12 months of follow-up were included. Articles combining PCL reconstruction with anterior cruciate ligament (ACL) reconstruction or other procedures were excluded. Studies that enhanced PCL reconstruction with cell therapies or experimental physiotherapy regimens were not suitable. Only articles which reported quantitative data under the outcomes of interests were considered for inclusion.

### Data extraction

Data extraction was performed by one author (A.P.). The following data at baseline were collected: author, year, journal, length of the follow-up, number of procedures, mean age of the patient age, percentage of women and type of autograft used. For each autograft, the following data were retrieved at last follow-up: Lysholm Knee Scoring Scale, International Knee Document Committee (IKDC), ROM, joint laxity measured by KT-1000 arthrometer, rate of revision and anterior knee pain.

### Methodology quality assessment

For the methodological quality assessment, the Coleman Methodology Score (CMS) was used.^25^ The CMS is widely used to evaluate the methodological quality of systematic reviews and meta-analyses and is highly reliable.^26–28^ This score allows for an analysis of the included papers based on several points of interest, including study size, follow-up duration, surgical approach, type of study, description of diagnosis, surgical technique and rehabilitation. Additional outcome criteria assessment, the procedures for assessing outcomes and the subject selection process were also evaluated. The CMS rates articles with values between 0 (poor) and 100 (excellent). Articles with values of >60 are considered to be satisfactory.

### Statistical analysis

The statistical analyses were performed by the main author (F.M.) using the STATA Software/MP (StataCorporation, College Station, TX, USA). For descriptive statistics, mean and standard deviation was used. For dichotomic data, the frequency was estimated. Continuous data were analysed using the analysis of variance. The Tukey Honestly Significant Difference *post hoc* test was also performed. The confidence interval (CI) was set at 95% in all the comparisons. Values of *P* < 0.05 were considered to be statistically significant.

## Results

### Search results

**Fig. 1:**
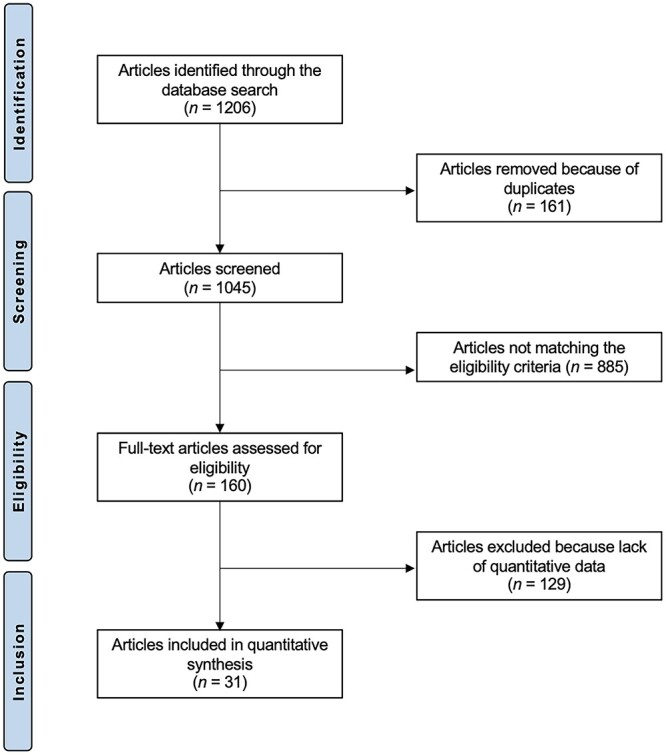
Flowchart of the literature search.

The initial literature search resulted in 1061 articles of which 361 were excluded because of redundancy. Another 650 articles were excluded because they did not match the eligibility criteria: other autografts, allografts, synthetic grafts (*N* = 203), comments, reviews, letters, notes, protocols, editorials, guidelines or registries (*N* = 301), biomechanical and/or cadaveric studies (*N* = 50), multiligaments reconstruction (*N* = 46), short duration of the follow-up (*N* = 13) and enhancing PCL reconstruction with other procedures (*N* = 37). A further 29 articles did not report quantitative data under the endpoints of interest. Thus, a total of 31 articles were eligible for this systematic review (Fig. 1).

### Methodology quality assessment

The study size and the duration of the follow-up were acceptable in most of the included studies. Surgical approach, diagnosis and rehabilitation were described in most articles. Outcome measures and timing of assessment were often defined, providing moderate reliability. General health measures were rarely reported. The average CMS for the articles was 68.9, attesting an acceptable quality of the methodologies for the included articles.

### Patient demographics

Data were retrieved for 946 patients, with a mean age of 28.1 ± 0.8 years and a mean follow-up of 40.1 ± 10.8 months. Study generalities and patient demographic at baseline are shown in Table 1.

**Table 1:** Patient demographics of the included studies (BPTB; PLT: peroneus longus tendon)

Author, year	Design	Autograft	Follow-up	Patients	Mean age	Female
			(months)	(*n*)	(mean)	(%)
Cooper et al. 2004^29^	Prospective	BPTB	39.4	16	28	24.4
				25		
Lin et al. 2013^12^	Retrospective	BPTB	51.6	25	26.8	32
Ahn et al. 2005^30^	Retrospective	Hamstring	35	18	30	16.6
Boutefnouchet et al. 2012^31^	Retrospective	Hamstring	49.2	15	25	0
Chan et al. 2006^15^	Prospective	Hamstring	40	20	29	25
Chen et al. 2002^32^	Prospective	Hamstring	26	27	27	33.3
Chen et al. 2006^33^	Prospective	Hamstring	54	52	31	32.7
Cury et al. 2017^16^	Retrospective	Hamstring	24	16	31	6.2
Deehan et al. 2003^34^	Prospective	Hamstring	40	31	27	7.4
Deie et al. 2015^13^	Retrospective	Hamstring	150	27	34	33.3
				13	32	15.4
Hagino et al. 2018^35^	Retrospective	Hamstring	24	23	28.9	27.7
Jackson et al. 2008^36^	Prospective	Hamstring	120	26	28	3.8
Jain et al. 2016^37^	Retrospective	Hamstring	28.1	22	27.4	0
				18	26.4	0
Li et al. 2014^38^	Retrospective	Hamstring	27.6	18	31.3	27.7
Li et. al 2008^39^	Retrospective	Hamstring	28.8	15	20–43	13.3
Lin et al. 2013^12^	Retrospective	Hamstring	51.1	34	26.2	21
Ma et al. 2019^40^	Prospective	Hamstring	28	60	33.6	30
Mestriner et al. 2019^41^	Retrospective	Hamstring	24	18		
Norbakhsh et al. 2014^42^	Prospective	Hamstring	42	52	27	19.2
Rhatomy et al. 2020^43^	Prospective	Hamstring	24	27	30.3	59.2
Saragaglia et al. 2019^44^	Retrospective	Hamstring	27	8	24.5	0
Sun et al. 2015^45^	Retrospective	Hamstring	37.2	36	31.1	25
			39.6	35	33.4	22.2
Tornese et al. 2008^46^	Randomized	Hamstring	12	7	24	14.2
Wang et al. 2017^14^	Retrospective	Hamstring	71.6	41	32	44.9
				17	32	44.8
Xu et al. 2014^47^	Retrospective	Hamstring	51	16	29.1	43.7
Zaho et al. 2007^48^	Retrospective	Hamstring	31.2	21	23–46	23.8
			30	22	19–45	18.1
Rhatomy et al. 2020^43^	Prospective	PLT	24	28	29.1	21.4
Setyawan et al. 2019^23^	Retrospective	PLT	24	15	25.9	26.6
Aglietti et al. 2002^21^	Prospective	Quadriceps	42	18	26.7	38.8
Chen et al. 1999^49^	Retrospective	Quadriceps	12–18	12	29	25
Chen et al. 2004^22^	Retrospective	Quadriceps	46	29	28	38
Wu et al. 2008^50^	Prospective	Quadriceps	66	22	27	22.7
Zayni et al. 2011^20^	Retrospective	Quadriceps	29	21	29	14.3

### Outcomes of interest

The BPTB group demonstrated the greatest mean Lysholm score (91.9 ± 6.7), followed by hamstring (88.5 ± 4.3), quadriceps (86.9 ± 4.6) and the peroneus longus tendon cohorts (81.7 ± 2.1) (Table 2).

**Table 2:** Results of the Lysholm score

Lysholm	BPTB	Hamstring	Peroneus	Quadriceps
BPTB	1			
Hamstring	MD: −3.4; 95% CI: −6.1 to −0.6; *P* = 0.005	1		
Peroneus	MD: −10.2; 95% CI: −12.9 to −7.4; *P* < 0.0001	MD: −6.8; 95% CI: −9.5 to −4.0; *P* < 0.0001	1	
Quadriceps	MD: −3.8; 95% CI: −6.5 to −1.0; *P* = 0.001	MD: −0.4; 95% CI: −3.1 to 2.3; *P* = 0.9	MD 6.4; 95% CI: 3.6–9.1; *P* < 0.0001	1

The BPTB group reported the lower mean instrumental laxity (2.8 ± 0.9), followed by the hamstring (3.2 ± 0.9) and quadriceps tendon groups (3.0 ± 1.0) (Table 3).

**Table 3:** Results of the mean instrumental laxity

Arthrometer	BPTB	Hamstring	Quadriceps
BPTB	1		
Hamstring	MD: 0.4; 95% CI: −0.1 to 0.9; *P* = 0.2	1	
Quadriceps	MD: 0.6; 95% CI: 0.0–1.1; *P* = 0.02	MD: 0.2; 95% CI: −0.3 to 0.7; *P* = 0.9	1

Patients undergoing PCL reconstruction using hamstrings exhibited the higher IKDC (82.8 ± 2.7), followed by the PTL (79.7 ± 2.2), BPTB (75.3 ± 1.6) and quadriceps (74.5 ± 3.1) tendon groups (Table 4).

**Table 4:** Results of the IKDC score

IKDC	BPTB	Hamstring	Peroneus	Quadriceps
BPTB	1			
Hamstring	MD: 7.5; 95% CI: 5.0–9.9; *P* < 0.0001	1		
Peroneus	MD: 4.4; 95% CI: 1.9–6.8; *P* < 0.0001	MD: −3.1; 95% CI: −5.5 to −0.6; *P* = 0.002	1	
Quadriceps	MD: 1.8; 95% CI: −0.6 to 4.2; *P* = 0.2	MD: −5.7; 95% CI: −8.1 to −3.2; *P* < 0.0001	MD: −2.6; 95% CI: −5.0 to −0.1; *P* = 0.02	1

Similarity was found in ROM between the BPTB and hamstring (MD: −1.1; 95% CI: −4.4–2.2; *P* = 0.8) autografts group (Table 5).

### Complications

The quadriceps tendon groups showed a rate of revision of 1.0% (1 of 102), and the hamstring showed 0.8% (6 of 755). No revision was experienced by any patients of the PLT and BPTB cohorts. Anterior knee pain was observed in 9.1% (6 of 66) of patients in the BPTB group, and this was observed in 7.0% (3 of 43) in the PTL group and in 1.0% (7 of 735) in the hamstring group. No anterior knee pain was experienced by patients in the quadriceps group. The complications related to each graft are shown in detail in Table 6.

**Table 5:** Results of the ROM

ROM	BPTB	Hamstring
BPTB	1	
Hamstring	MD: −1.1; 95% CI: −4.4 to 2.2; *P* = 0.8	1

**Table 6:** Analysis of complications

Variable	BPTB	Hamstring	Peroneus longus	Quadriceps
Revision	0% (0 of 66)	0.8% (6 of 755)	0% (0 of 43)	1.0% (1 of 102)
Anterior knee pain	9.1% (6 of 66)	1.0% (7 of 735)	7.0% (3 of 43)	0% (0 of 102)

## Discussion

PCL reconstruction using an autologous ipsilateral BPTB graft and hamstring likely represents the most suitable graft for primary isolated PCL reconstruction. BPTB demonstrated the greater Lysholm score and the lower joint laxity at arthrometer. Hamstring produced the higher IKDC score. BPTB and hamstring evidenced similar ROM. BPTB and PLT are associated with the highest rate of anterior knee pain.

Hamstring is the most common autograft employed for cruciate ligament reconstruction.^12–16^^,^^30–48^ Compared to BPTB and quadriceps autografts, hamstring grafts are associated with less morbidity, especially with regard to anterior knee pain during kneeling and extension deficit.^39^ In addition, the harvest of hamstring autografts is associated with greater posterior stability compared to BPTB.^12^^,^^51^^,^^52^ Following adequate rehabilitation, no decrease in hamstring muscle strength should be expected.^53^ On the other hand, hamstring autografts may have disadvantages, including their small size, the high risk of saphenous nerve injury, thigh hypotrophy and pain along the hamstring region.^32^^,^^54–56^ From a biomechanical point of view, hamstring autografts demonstrated less stiffness than the native PCL along with decreased flexion and internal rotation strength of the knee.^32^^,^^54–56^

PCL reconstruction with BPTB allows fast return to sport and enables bone-to-bone healing in ~4–6 weeks.^12^^,^^18^ A biomechanical comparison of tibial inlay and tibial tunnel techniques for PCL reconstruction using BPTB grafts demonstrated that both techniques result in significant greater strength than that measured in the native PCL with the knee flexed beyond 85°.^57^ Posterior tibial translation between BPTB and hamstring PCL reconstruction was compared under 100-N cyclic loading in a cadaveric study^58^; the hamstring group demonstrated greater laxity than BPTB.^58^

Quadriceps tendon autograft is a viable alternative for PCL reconstruction. Patients treated with a quadriceps tendon autograft reported satisfactory clinical outcomes, with optimal knee stability and quick return to preinjury level of activity.^20^ The quadriceps tendon is thicker, longer and wider than the patellar tendon, demonstrating sufficient size and strength for PCL reconstruction.^49^^,^^59^ The ultimate tensile failure load for quadriceps complexes occurred at 2173 ± 618 N compared with 1953 ± 325 N of the BPTB.^59^ However, in a cadaveric study, quadriceps and BPTB autografts demonstrated similar load to failure, no difference in load to failure stiffness and displacement at failure.^60^

PLT autografts are recommended for athletes who require dominant hamstring strengths to reduce the low incidence of anterior knee pain and kneeling pain.^23^ PLT autografts have been used in ACL reconstruction with minimal donor site morbidity, good clinical outcomes and tensile strength compared to hamstring autografts.^61^ Previous studies demonstrated that PCL reconstruction using PLT autograft showed good functional outcome at 2-year follow-up.^23^^,^^62^

Several studies have compared the clinical outcomes of PCL reconstruction with autograft versus allograft and have demonstrated no significant differences in outcomes.^12^^,^^14^^,^^30^^,^^38^^,^^40^^,^^45^^,^^63–66^ Although autografts produce comparable results to allografts, the use of allografts can eliminate donor site morbidity and minimize operative trauma.^45^^,^^67^ However, complications such as tissue rejection and delayed revascularization are a concern.^30^ To overcome these complications, the Ligament Advanced Reinforcement System has been introduced with satisfying clinical outcomes.^39^^,^^44^^,^^47^^,^^68–71^

The present study has several limitations. The design of the studies included for analysis was mostly prospective and retrospective, and only one randomized controlled trial was included. The limited study size along with the heterogeneous inclusion eligibility criteria were other important sources’ bias of the present study. The analyses were conducted irrespective of the type of the technique used for reconstruction (double or single bundle). The limited number of samples included for analysis may have jeopardized the reliability of these results. Thus, given these limitations, data must be interpreted with caution. Strengths of the present work were the comprehensive nature of the literature search along with the strict eligibility criteria and the adequate baseline comparability. The timing of the evaluation of the results was clearly indicated by most of studies. Most studies used outcome criteria with good reliability. The selection criteria were often reported and unbiased. Future high-quality studies involving a larger number of patients and longer follow-up are required to detect less common complications.

## Conclusion

The BPTB may represent the most suitable tendon for primary isolated PCL reconstruction. Further clinical investigations are required to infer solid conclusions.

## Conflict of interest statement

The authors have no potential conflicts of interest.

## Ethical approval

This article does not contain any studies with human participants or animals performed by any of the authors.

## Informed consent

For this type of study, informed consent is not required.

## Data availability statement

The data underlying this article are available in the article and in its online supplementary material.
